# Adaptive optics in super-resolution microscopy

**DOI:** 10.52601/bpr.2021.210015

**Published:** 2021-08-31

**Authors:** Jingyu Wang, Yongdeng Zhang

**Affiliations:** 1 Department of Engineering Science, University of Oxford, Oxford, OX1 3PJ, UK; 2 School of Life Sciences, Westlake University, Hangzhou 310024, China; 3 Westlake Laboratory of Life Sciences and Biomedicine, Hangzhou 310024, China

**Keywords:** Adaptive optics, Optical aberrations, Aberration correction, Wavefront sensor, Deformable mirror, Super-resolution microscopy

## Abstract

Fluorescence microscopy has become a routine tool in biology for interrogating life activities with minimal perturbation. While the resolution of fluorescence microscopy is in theory governed only by the diffraction of light, the resolution obtainable in practice is also constrained by the presence of optical aberrations. The past two decades have witnessed the advent of super-resolution microscopy that overcomes the diffraction barrier, enabling numerous biological investigations at the nanoscale. Adaptive optics, a technique borrowed from astronomical imaging, has been applied to correct for optical aberrations in essentially every microscopy modality, especially in super-resolution microscopy in the last decade, to restore optimal image quality and resolution. In this review, we briefly introduce the fundamental concepts of adaptive optics and the operating principles of the major super-resolution imaging techniques. We highlight some recent implementations and advances in adaptive optics for active and dynamic aberration correction in super-resolution microscopy.

## INTRODUCTION

Fluorescence microscopy is one of the most straightforward and effective approaches to observe the intrinsic processes of life activities and reveal their differences and variations in a non-invasive manner. When exploring the underlying molecular mechanisms at the subcellular level, the resolution of the imaging technique largely determines the depth of the studies. Super-resolution microscopy, representing a range of fluorescence microscopy techniques with different principles, breaks the diffraction limit and bridges the resolution gap between traditional optical microscopy and electron microscopy, promoting biological research at the nanoscale (Sahl *et al*. 2017; Schermelleh *et al*. 2019; Sigal *et al*. 2018).

For conventional microscopes, the obtainable resolution is constrained not only by the diffraction of light but also by the presence of optical aberrations. Super-resolution microscopes, pursuing perfect images with a molecular-level resolution, are more sensitive to optical aberrations. In super-resolution microscopy, optical aberrations distort the point spread function (PSF) of the imaging systems in three dimensions, thereby reducing the image contrast and resolution. In some scenarios, aberrations can even eliminate any resolution improvements brought by the super-resolution techniques. Furthermore, optical aberrations reduce the efficiency of light delivery and collection, thus decreasing the signal-to-noise ratios. In most cases, the magnitude of optical aberrations grows substantially with increasing imaging depth, thus becoming a formidable obstacle for deep-tissue imaging. To address these challenges, adaptive optics (AO), a technique widely used in astronomy, has been introduced to correct for optical aberrations and restore the image quality and resolution in microscopy, including super-resolution microscopy (Booth 2014; Booth *et al*. 2015). The basic idea of AO is to compensate for the estimated optical aberration by adding an equal but opposite amount of distortion to the wavefront of the imaging system through an active wavefront shaping device.

In the past decade, aberration correction in microscopy with AO has proven to be a tractable solution and has been applied to essentially all microscope modalities (Ahn *et al*. 2019; Ji 2017; Rodriguez and Ji 2018). In this review, we focus on the advances and applications of AO in super-resolution microscopy techniques. We briefly introduce the fundamental concepts and principles of AO as well as its implementations in super-resolution microscopy.

## FUNDAMENTALS OF ADAPTIVE OPTICS

In this section, we introduce several fundamental but important concepts about adaptive optics.

### Optical aberrations

**Figure 1:**
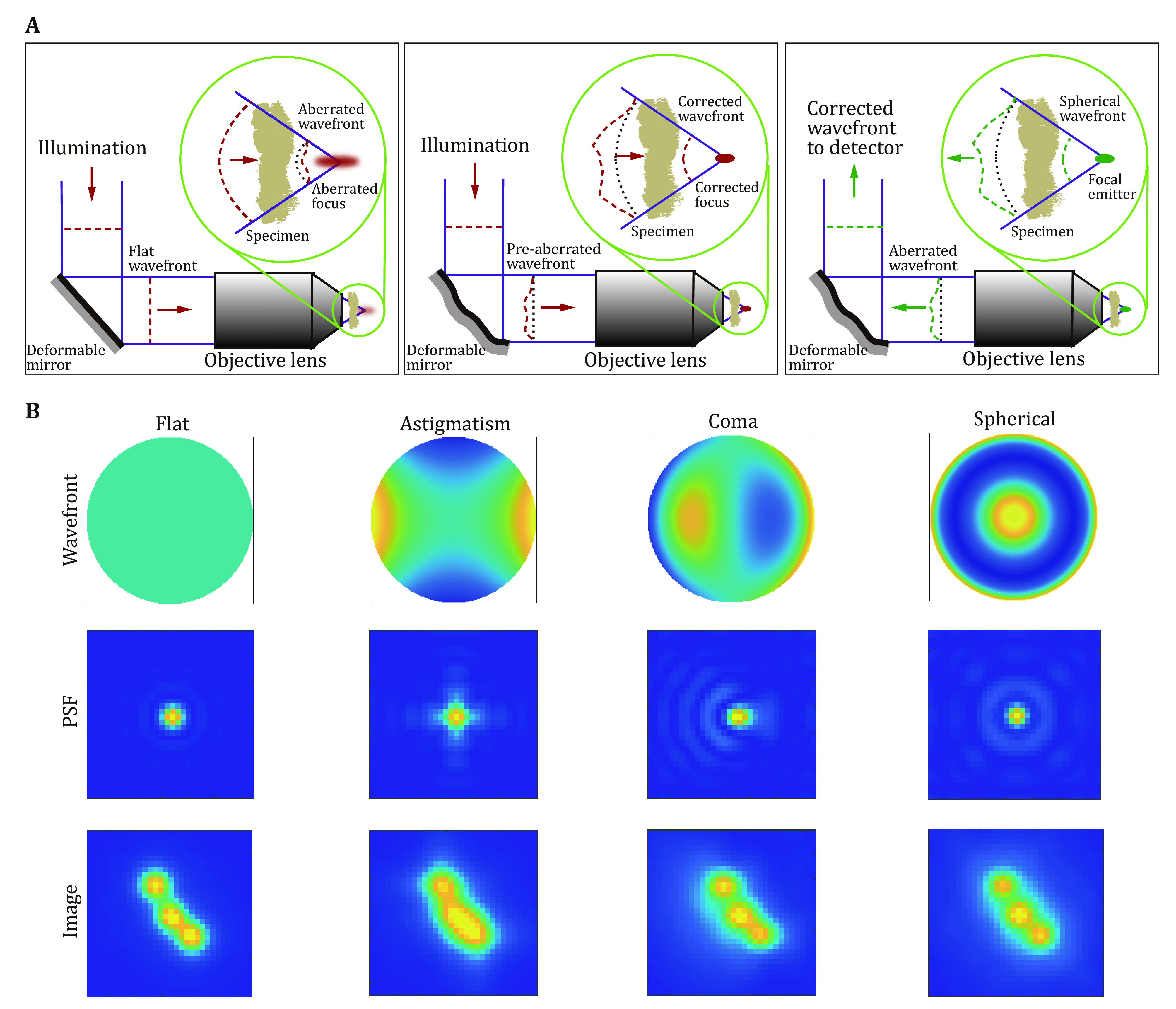
Optical aberration and correction in fluorescence microscopy. **A** The wavefront is distorted by sample-induced aberrations. The aberrations exist in both illumination (excitation) and detection (imaging) paths and can be corrected by deformable mirrors. Adapted from Booth (2014). **B** Examples showing the effect of three common aberration modes

In fluorescence microscopes, light from a point emitter, propagating in the form of a spherical wavefront (phase), is firstly collected by the objective lens and converted into a planar wavefront, then converges as a spherical wavefront after the tube lens, which eventually forms an ideal diffraction-limited spot (so-called PSF) at the detector. Aberrations are imperfections that cause the light to deviate from the ideal optical path, distorting the wavefront and smearing the focus, thereby reducing the image quality and resolution. In fluorescence microscopes, the aberrations shared by the illumination and detection paths can be corrected simultaneously in the common beam path (Fig. 1A). The phase information at the back pupil plane of the objective lens is commonly used to quantify the aberrations. In particular, phase aberrations can be analyzed by decomposing the pupil function into Zernike polynomials, which are an infinite set of orthogonal polynomials defined over the unit disk (normalized back aperture in microscopy) (Mahajan 1994). Zernike polynomials are closely related to classic aberrations such as spherical, coma, and astigmatism, making them the most popular and convenient choice for aberration representation (Fig. 1B).

Both optical systems and biological samples can introduce aberrations. System-induced aberrations arise from the fact that optical components are non-ideal and suffer from manufacturing defects. For example, dichroic beamsplitters or mirrors may introduce a certain degree of aberrations (mainly astigmatism) if the surface is not sufficiently flat. Additionally, misalignments of the optical system can generate different kinds of aberrations. For example, making the beam not go through the lenses at the center co-axially or having a slight tip/tilt between the objective and the coverslip can introduce coma. In general, system-induced aberrations are relatively stable over time and can be minimized by using high-quality optical components and careful alignment. Sample-induced aberrations, however, are much more difficult to deal with. They can arise from the refractive index mismatch between the objective immersion medium and the samples, primarily appearing as spherical aberrations. Moreover, heterogeneity within biological samples can generate complex aberrations that vary among samples and even among different regions of the same sample. While system-induced aberrations can be well characterized using calibration samples (fluorescent beads embedded in agarose, *etc*.), aberrations from biological specimens are almost unpredictable in practice. Therefore, when imaging thick samples, sample-induced aberrations are arguably the most prominent aberration source and are also much more challenging to correct.

### Aberration measurement and correction

The first step which is also the key step in AO correction is aberration measurement. Generally, there are two main approaches: direct or indirect wavefront measurement.

In direct wavefront measurement or direct wavefront sensing, a wavefront sensor (WFS) is used to directly measure the phase aberration from the received wavefront. The Shack–Hartmann wavefront sensor (SH-WFS) is most commonly used due to its compact size, low cost, simple structure, and easy operation. A SH-WFS normally consists of a 2D microlens array that segments wavefront into sub-apertures and focuses them onto individual spots on a camera. A perfect wavefront forms a uniformly distributed spot pattern, while any aberration causes lateral shifts of the spots. Therefore, the aberrations in the wavefront can be extracted from the displacements of the spots from the non-aberrated positions. Typically, a guide star (a point-like light source) is required. There are two popular approaches to generate such guide stars in microscopy. The first is to use the fluorescent signal generated at the focal spot of two-photon excitation. Guide stars can also be generated by using fluorescent beads or gold nanoparticles with sizes well below the diffraction limit. Ideally, aberration information can be obtained from a single measurement, allowing for high-speed AO correction. However, the implementation of a SH-WFS adds complexity and extra cost to the optical system and guide stars are not always available. Moreover, the addition of a WFS introduces non-common path aberrations that cannot be corrected easily (Sulai and Dubra 2014). Due to the lack of effective guide stars, direct wavefront sensing is not convenient for many widefield microscopes and is more preferable in laser scanning systems.

As early adopters of AO techniques, astronomical and ophthalmological imaging typically implement the direct wavefront sensing approach in a closed-loop scheme, so that aberrated light is measured by the WFS after passing through the AO device. This approach allows incremental improvement of the wavefront correction over iterations, yielding a robust wavefront correction performance. However, close-loop AO requires splitting a fraction of the fluorescence signal for wavefront sensing, which is not ideal for scenarios with low photon budgets, such as single-molecule imaging. In many microscopy studies, aberrations are either static or slow-changing during data acquisition, so close-loop AO is not necessary. However, if aberrations in the sample or system vary temporally or spatially, direct wavefront sensing and close-loop correction are highly desirable.

**Figure 2:**
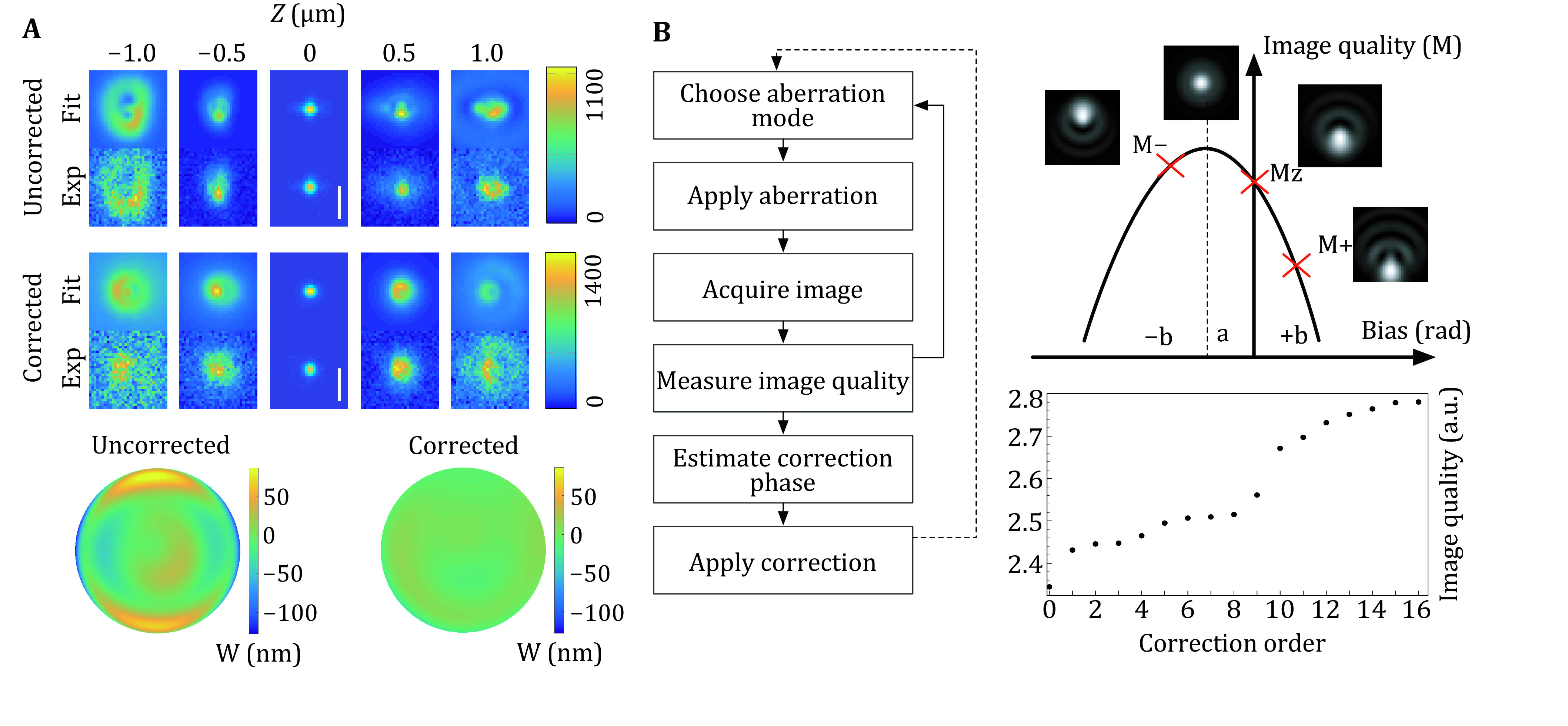
Indirect wavefront measurement. **A** Aberration measurement and correction by phase retrieval. Scale bar, 1 μm. Modified from Siemons *et al*. (2018). **B** Image quality metric-based wavefront sensing. Adapted from Zurauskas *et al*. (2019)

Indirect wavefront measurement or indirect wavefront sensing is a sensorless approach that estimates the aberrations indirectly from the images produced by the microscope. Sensorless AO can be implemented in different ways (Wright *et al*. 2005). One approach is to use phase retrieval to reconstruct the pupil function by imaging fluorescent beads and aberrations can be extracted from the pupil function and corrected afterwards (Fig. 2A). This approach is simple and useful for correcting system-induced aberrations. Another sensorless approach, based on image quality metrics, requires recording a series of images while intentionally applying aberration bias for each mode. The optimal amplitude of each aberration mode is determined by maximizing the image quality metric (brightness, contrast, sharpness, and resolution *etc*.). The procedure needs to be run iteratively for each aberration mode until the desired image quality is reached (Fig. 2B). This metric-based approach is conceptually compatible with any type of microscope and can be used for correcting sample-induced aberrations. With carefully chosen metrics, sensorless approaches can yield comparable performance with direct wavefront sensing in many situations (Wahl *et al*. 2019). However, correction speed is the main limitation as N aberration modes require at least N + 1 measurements (typically 2N + 1). This is not necessarily problematic when aberrations in the samples are relatively static over the timescale of imaging. Thus, sensorless AO approaches have been commonly used in the field of super-resolution microscopy.

### Adaptive optical devices

The simplest adaptive optical device in microscopy is the objective correction collar, which allows correction for spherical aberrations induced by varying thicknesses of the coverslip, distance between the sample and the coverslip, or temperature changes. Many high-end objectives are now equipped with manual correction collars, and some offer motorized correction collars as an option.

AO correction requires active wavefront shaping devices that can perform complex wavefront modulations in the conjugated pupil plane of the objective lens. These devices need to have enough degrees of freedom to compensate for complex aberrations. In general, there are two main optical devices used for active aberration corrections: deformable mirrors (DMs) and liquid crystal spatial light modulators (SLMs). Although they share the same purpose, *i*.*e*. to compensate for optical aberrations by adding an equal but opposite shape to the aberrated wavefront, their different architectures and characteristics make them suitable for different applications.

DMs are wavefront control devices widely used in astronomy to compensate for aberrations due to atmospheric turbulence. The DM surface can be either continuous or segmented. Continuous DMs usually have a thin membrane coated with a reflective metal layer. The membrane can be shaped by a number of electrically controlled actuators. For AO correction in microscope systems with prominent low-order aberration modes, a continuous surface is usually preferred. The stroke of continuous DMs ranges from a few micrometers to tens of micrometers. Large strokes are useful for correcting extreme aberrations or remote focusing but may suffer from drift and hysteresis. On the contrary, in segmented DMs, a single or small number of actuators control certain degrees of freedom of a miniature reflective surface. The coupling between adjacent actuators is minimal or none for segmented DMs, making them more suitable for generating high-order aberration modes. The actuators of DMs can be made based on the magnetic, micro-electromechanical system (MEMS), electrostatic electrodes, and piezoelectric devices.

Thanks to the reflective nature of the metal coating, DMs are insensitive to polarization and wavelength, meanwhile providing high optical efficiency. This is particularly important as the fluorescence is unpolarized and broadband. Thus, DMs are widely used in fluorescence microscopes that are often designed for multiple illumination and detection wavelengths. When employed in the common path, a single DM is sufficient to correct for aberrations in both illumination and detection beam paths. However, due to coupling between adjacent actuators and manufacturing imperfections, a calibration or training step is usually necessary before a DM can be used for accurate wavefront control, especially in sensorless AO. The calibration can be done by using a wavefront sensor, either an interferometer (Antonello *et al*. 2020b) or a SH-WFS, *in situ* or *ex situ*.

SLM is another popular device used to modulate the wavefront of light in AO systems. In general, SLMs are devices that can manipulate properties of light, including amplitude, phase, and polarization. The most common types of SLMs are built on an array of cells of liquid crystal on silicon. They usually have a large number of cells (pixels) that can modulate the phase of the incident light over a range of at least 2Pi individually. This type of SLMs is typically used for phase-only correction. The large number of pixels provides great flexibility in phase correction and manipulation. For example, a single SLM device can be spatially divided into multiple windows and used as multiple AO devices using a multi-pass configuration (Lenz *et al*. 2014). Phase wrapping techniques can be used to increase the range of phase modulation (Hacker *et al*. 2003). Although SLMs can be used for certain applications without precise calibration, recent studies have shown that pixel-wise calibration is as important as careful alignment for optimal performance (Dai *et al*. 2019; Siemons *et al*. 2018).

In contrast to DMs, SLMs are sensitive to polarization and wavelengths. Therefore, they are mainly used for phase modulation in the illumination path, although they can also be used in the detection path for a single wavelength band at the cost of half fluorescence. Compared to DMs, SLMs have much more actuators (~100,000 vs ~100) that can generate high-order wavefront aberrations but at much lower refresh rates (~100 Hz vs >2 kHz). It is worth noting that both DMs and SLMs should be placed at the plane conjugated to the back pupil of the objective lens, and their effective working aperture should match the pupil size for best performance. Since all DMs and most SLMs are used in a reflection configuration, it is common to have a small angle between the incident and reflected beam, making the effective projection of the beam slightly elliptical rather than circular. For this reason, in practice, the angle should not be too large (typically less than 15 degrees), and the elliptical projection effect can be handled by calibrations. While both DM and SLM are typically implemented as reflective devices in AO systems, transmissive devices, such as adaptive lenses or liquid lenses, have recently become available for microscopy, which allow for easier integration of AO optics to existing microscope systems (Banerjee *et al*. 2018; Chiu *et al*. 2012; Pozzi *et al*. 2020).

## ABERRATION CORRECTION IN SUPER-RESOLUTION MICROSCOPY

Super-resolution microscopy has revolutionized biological imaging over the past two decades. However, higher resolutions make it highly dependent on the optimal performance of the imaging systems and thus more susceptible to optical aberrations. In this section, we briefly describe the basic principles of three main super-resolution imaging techniques and the implementation of adaptive optics in these approaches.

### Single-molecule localization microscopy

Single-molecule localization microscopy (SMLM) is an umbrella term for a series of methods that share the same operating principles such as (F) PALM, (d) STORM, GSDIM, and PAINT (Baddeley and Bewersdorf 2018; Möckl and Moerner 2020; Sauer and Heilemann 2017). In conventional fluorescence microscopy, all fluorophores emit photons simultaneously, and their PSFs overlap with each other, forming a diffraction-limited image. The key of SMLM is to separate molecules with overlapped PSFs in time rather than in space. That is, at each time point, only a few emitters are switched to the “on” state and become visible (or show detectable signals over the background as in PAINT), while most of the remaining emitters stay in the “off” state. The fluorescence signal from the emitters is typically recorded by a camera. This on-off switching (so-called blinking) cycles for thousands of frames until most fluorophores are photobleached or the desired localization density is reached. The on/off contrast is critical and to a great extent determines the resolution of SMLM. During data processing, emitters at each frame are identified and localized with nanometer precision and eventually combined to render a super-resolution image. The attainable resolution of SMLM depends on how well we can estimate the positions of the emitters from the emission PSFs. It has been shown that optical aberrations have a strong impact on the resolution of SMLM (Coles *et al*. 2016; Deng and Shaevitz 2009).

SMLM is normally based on widefield illumination. The illumination profile typically follows a Gaussian distribution but can be shaped into a uniform distribution. The illumination intensity can affect the blinking properties of fluorophores but doesn’t introduce aberrations. Thus, aberration correction is only necessary for the emission path, and DMs are often used for this purpose due to their zero chromatic aberration and insensitivity to polarizations, although SLMs have been used for PSF engineering and aberration correction in SMLM (Siemons *et al*. 2018; Wang *et al*. 2018).

**Figure 3:**
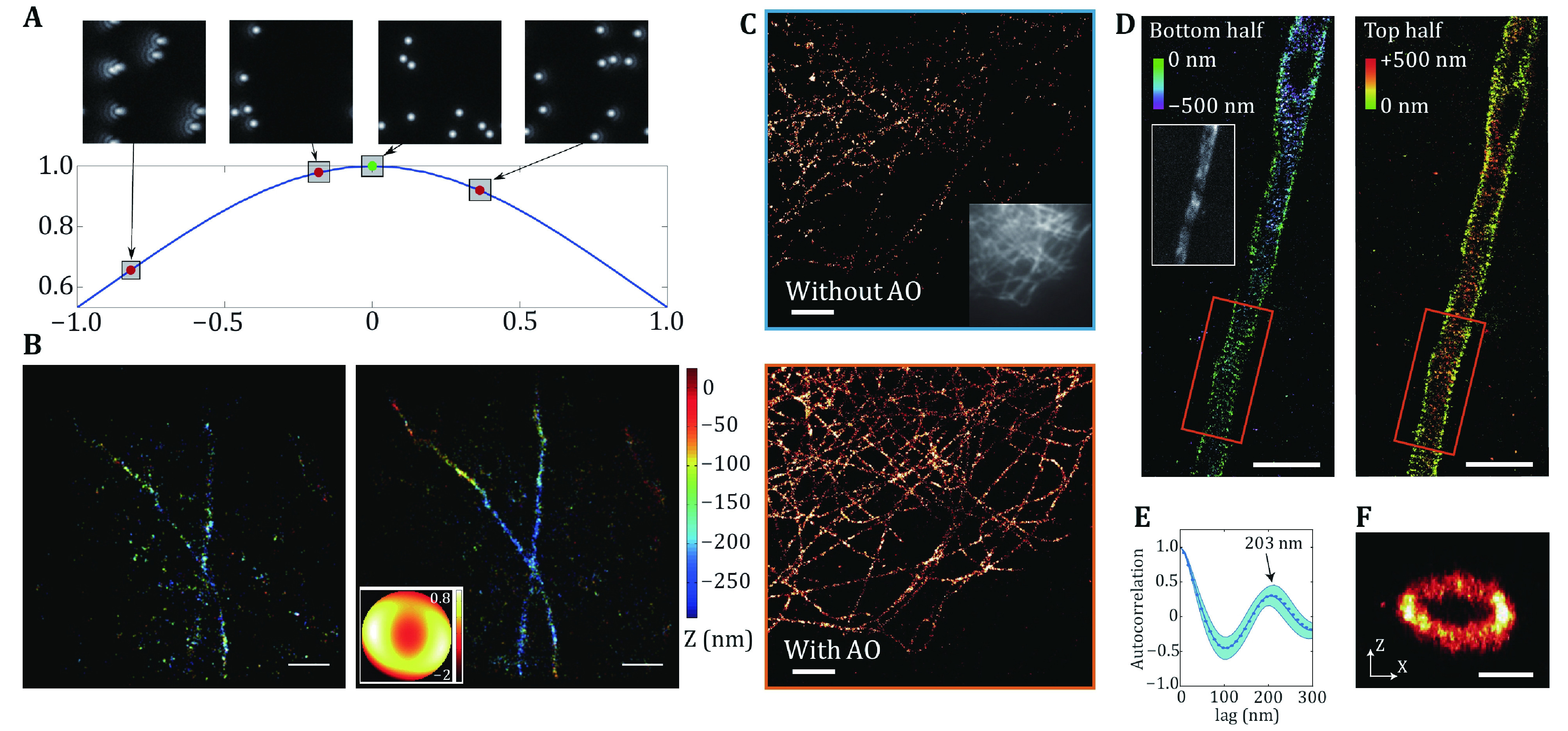
Aberration correction in SMLM. **A** Principle of metric-based aberration measurement. **B** 3D-STORM images of microtubules with and without aberration correction. Scale bar, 1 μm. **A** and **B** are both adapted from Burke *et al*. (2015). **C** SMLM reconstruction of microtubules in COS-7 cells through a 50-μm thick brain section without/with AO correction. Inset shows the widefield image before correction. Scale bar, 2 μm. **D** SMLM reconstruction of a layer 5 pyramidal neuron AIS stained for V-spectrin in a rat brain slice at 50-μm depth. Scale bar, 2 μm. **E** The average autocorrelation shows a clear periodicity with the peak at 203 nm. **F** Cross-section of the rectangular area indicated in **D**. Scale bar, 500 nm. **C–F** are adapted from Siemons *et al*. (2021)

Aberration correction in SMLM by DMs is relatively simple and straightforward, but aberration estimation is much trickier. While system-induced aberrations can be corrected in a predictable manner (Izeddin *et al*. 2012), correction for sample-induced aberrations is not trivial. Luckily, the blinking images of SMLM are essentially the convolution of the emitters with the system PSF, making it possible to estimate the optical aberrations directly from the raw images. Thus, aberration correction can be performed during image acquisition. Burke *et al*. designed a sensorless AO scheme capable of performing feedback correction for sample-induced aberrations on a dSTORM microscope (Burke *et al*. 2015). They established an image-based metric in Fourier space (M1) and estimated the aberrations from the first few hundreds of blinking images and then performed the model-based correction through the rest of the data acquisition (Fig. 3A and 3B). Another group combined an intensity-insensitive Fourier Metric with a genetic algorithm to correct for the aberrations and optimize the PSFs in real-time (Tehrani *et al*. 2015). However, this approach requires a few thousand frames to converge as it needs random mutation to avoid local minima. Therefore, they adopted a particle swarm optimization algorithm (M2) to speed up the convergence procedure by an order of magnitude (Tehrani *et al*. 2017). Mlodzianoski *et al*. developed an approach that combines adaptive PSF shaping with an efficient sensorless AO method based on simplex optimization (M3) to allow robust volumetric 3D imaging through thick specimens (Mlodzianoski *et al*. 2018). Siemons *et al*. systematically compared the three metrics mentioned above (M1, M2 and M3) and found M3 was able to achieve consistent correction (Siemons *et al*. 2021). They further improved the M3 metric by combining it with model-based optimization to robustly correct aberrations in realistic signal and noise levels up to a depth of 50 μm in tissue (Fig. 3C–3F).

As the final super-resolution image is constructed from localizations in SMLM, aberrations can also be handled offline during post-processing in the absence of active adaptive optical elements. Several groups have implemented different strategies to deal with depth-dependent aberrations (spherical, coma, *etc*.) (Cabriel *et al*. 2018; Carlini *et al*. 2015; McGorty *et al*. 2014) or field-dependent aberrations (von Diezmann *et al*. 2015), but other more complex aberrations require more sophisticated algorithms. One approach is to use an experimental PSF model that contains system-specific aberrations instead of a theoretical one (like Gaussian) (Li *et al*. 2019). Alternatively, Liu *et al*. reported a procedure to retrieve the pupil function from fluorescent beads images and generate PSFs for accurate 3D single-molecule localization (Liu *et al*. 2013). Although their approach can also be modified to include depth-dependent aberrations, it can not account for sample-induced aberrations due to heterogeneity within the specimen. To address this challenge, Xu *et al*. proposed a novel phase retrieval strategy (INSPR) that enables the construction of an *in situ* 3D PSF of single emitters directly from single-molecule blinking images (Xu *et al*. 2020). They further demonstrated that their approach can correct for both system- and sample-induced aberrations, thus resolving ultrastructures within whole-cell and tissues with high resolution and fidelity. In principle, aberrations estimated from the blinking images by INSPR can be immediately corrected by the DM, producing distortion-free raw images for subsequent data analysis.

### Structured illumination microscopy

Structured illumination microscopy (SIM) is another widefield-based super-resolution approach that theoretically doubles the resolution of a fluorescence microscope with standard fluorescent probes (Heintzmann and Huser 2017; Prakash *et al*. 2021; Wu and Shroff 2018). In the most common type of SIM, sinusoidal stripe illumination patterns at different orientations and phases are generated to illuminate the sample, which shifts higher spatial frequency information of the structure into the observable region of the microscope. To cover the entire expanded optical transfer function (OTF) range, nine images (three phases and three structure illumination orientations) are typically required to reconstruct a super-resolution image in 2D-SIM, while 15 images (five phases and three orientations) are required for 3D-SIM. Since only a small number of acquisitions are required, SIM provides a good balance between spatial and temporal resolution. Higher resolution can also be achieved in saturated SIM (SSIM) or nonlinear SIM (NLSIM) but at the cost of lower imaging speeds and higher phototoxicity.

**Figure 4:**
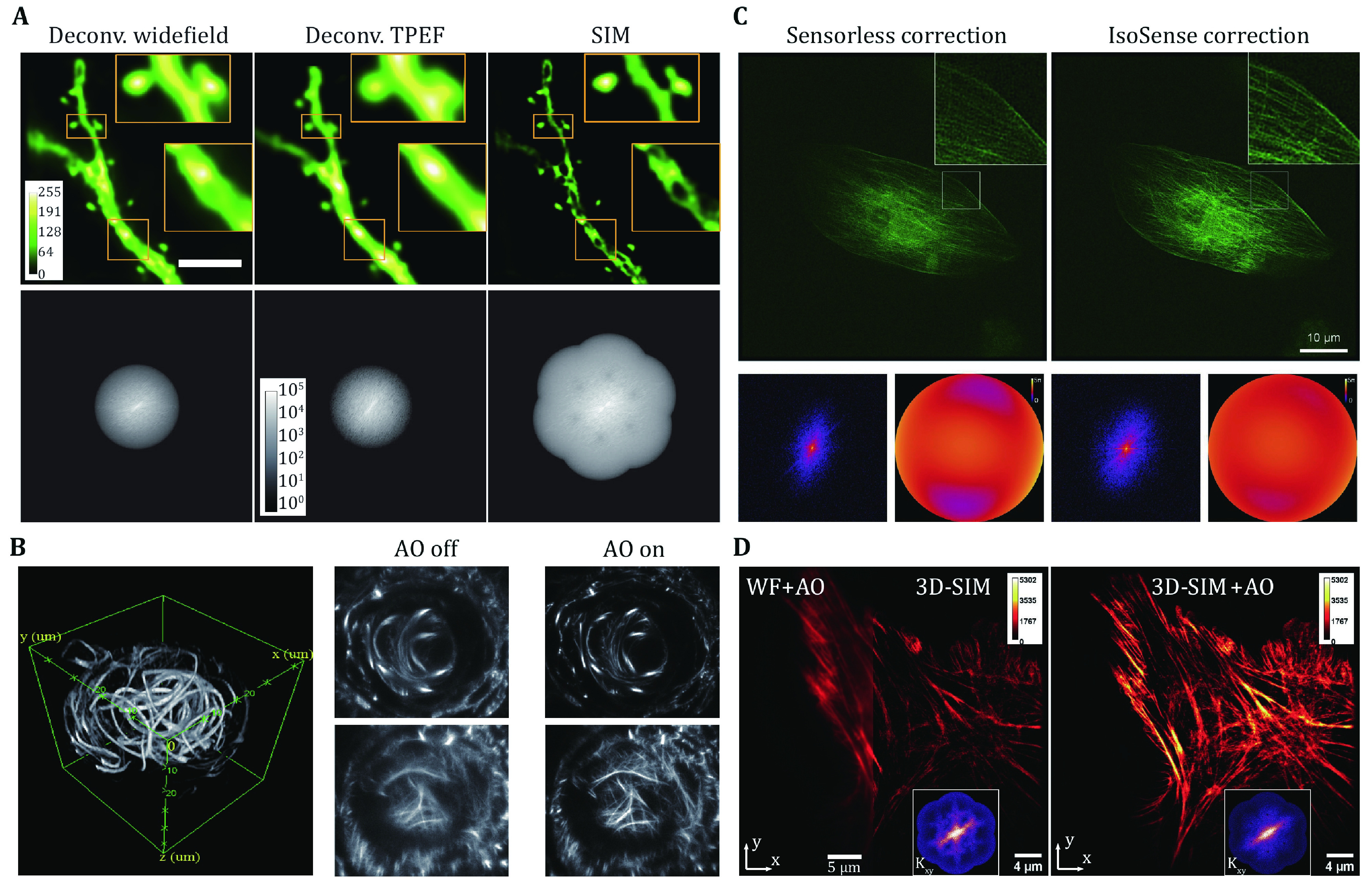
Aberration correction in SIM. **A** Images and corresponding OTFs of the same dendritic structure in a brain slice at a depth of 25 μm obtained with different imaging modalities, all with AO. Scale bar, 5 μm. Adapted from Turcotte *et al*. (2019). **B** 2P-ISIM images of microtubules in lens of embryonic zebrafish, with/without AO. Adapted from Zheng *et al*. (2018). **C** SIM images of microtubules with sensorless or IsoSense correction. Adapted from Zurauskas *et al*. (2019). **D** Image and corresponding OTFs of actin with different imaging modalities. Adapted from Lin *et al*. (2021)

Unlike SMLM, the illumination profile in SIM is critical as the image is essentially the multiplication of the sinusoidal stripe pattern and the distribution of fluorophores in the target structure. Aberrations in the excitation beam path can distort and smear the illumination pattern, thereby reducing image contrast and resolution, introducing artifacts, and even causing complete failure of image reconstruction (Arigovindan *et al*. 2012; Liu *et al*. 2020). Therefore, aberration corrections are required in both illumination and detection beam paths, which can be performed simultaneously by using a single DM in the common beam path. Both direct and indirect wavefront sensing methods have been applied to SIM to correct for optical aberrations and improve image quality. Turcotte *et al*. implemented a direct wavefront sensing module using multiphoton guide stars to facilitate super-resolution imaging of the brains in live zebrafish larvae and mice (Turcotte *et al*. 2019). They observed the dynamics of dendrites and dendritic spines at nanoscale resolution with the help of AO to correct for sample-induced aberrations (Fig. 4A). Similarly, Zheng *et al*. used a nonlinear guide star in two-photon instant SIM (2P-ISIM) to measure optical aberrations in both excitation and emission baths and correct them by a DM (Zheng *et al*. 2018). They demonstrated up to 40-fold intensity enhancement and substantial resolution recovery in cells and tissues at depths up to 250 μm (Fig. 4B). Using an indirect wavefront sensing method, Debarre *et al*. investigated the effect of different aberration modes on the illumination patterns in SIM and corrected for each mode independently using an image quality metric (Debarre *et al*. 2008). Thomas *et al*. introduced a phase retrieval approach to correct for aberrations in SIM, which improved the image contrast of fluorescent beads and achieved a resolution of 140 nm through 35 μm of tissue (Thomas *et al*. 2015). Zurauskas *et al*. reported a sensorless AO strategy based on image quality with improved sensitivity and reliability for aberration correction in 2D-SIM (Zurauskas *et al*. 2019). They combined it with a customized illumination pattern to enhance the sampling of OTF, producing more isotropic and better overall correction results (Fig. 4C). Lin *et al*. further extended the sensorless approach to 3D-SIM to recover information severely distorted by optical aberrations and to restore image quality and resolution when imaging a variety of biological samples (Fig. 4D) (Lin *et al*. 2021).

### Stimulated emission depletion microscopy

Stimulated emission depletion (STED) microscopy is a point-scanning approach that uses a nonlinear saturation process to induce transitions between the on and off states (Blom and Widengren 2017; Egner *et al*. 2020; Vicidomini *et al*. 2018). A STED microscope is essentially adding a depletion laser to a confocal microscope. The off-switching is done by the depletion laser that features an intensity minimum (ideally zero) at the focus. In 2D-STED, the depletion laser creates a doughnut-shaped focus by using a vortex phase mask. To produce a depletion focus in 3D-STED, a top-hat phase mask is used to deplete the fluorescence above and below the focal plane in addition to the vortex phase mask. In STED, only fluorophores in the focus center where the depletion laser shows zero intensity minima are allowed to emit detectable fluorescence (normal emission) while all other fluorophores under the excitation focus are subjected to stimulated emission. Thus, the effective PSF size (*i*.*e*. resolution) is determined by the width of the central intensity minima at the depletion focus rather than the excitation focus.

**Figure 5:**
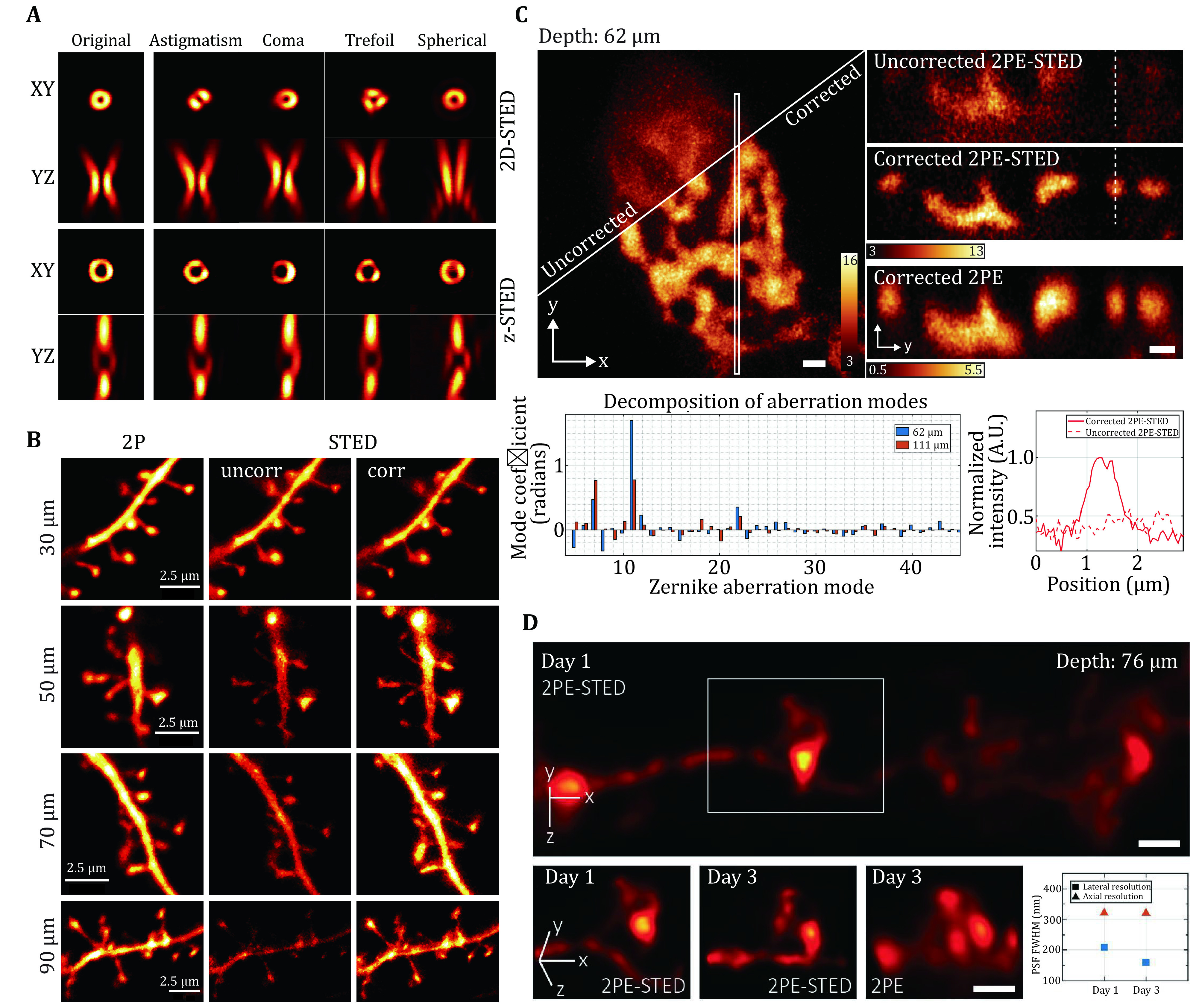
Aberration correction in STED. **A** Effect of typical aberrations in STED. **B** STED images of dendritic segments in hippocampal acute slices at various depths. **A** and **B** are both adapted from Bancelin *et al*. (2021). **C** Images of H2B-GFP within the nucleus located 62 μm below the tissue surface with different imaging modalities. Scale bars, 1 μm. **D** 2PE-STED images of a dendrite 76 μm below the cortical surface with AO correction. Scale bar, 1 μm. **C** and **D** are adapted from Velasco *et al*. (2021)

A STED microscope consists of three independent beam paths, excitation, emission, and depletion, all of which suffer from optical aberrations and therefore require correction. Among the three beam paths, the profile of the depletion beam is arguably the most critical one, and it is sensitive to many types of aberrations (Fig. 5A) (Antonello *et al*. 2016; Antonello *et al*. 2017; Deng *et al*. 2009, 2010). In general, aberration modes that distort the focal distribution but maintain zero intensity will reduce the STED resolution towards the confocal level. In contrast, aberration modes that destroy the zero intensity will deplete most fluorescence with little or no improvement in resolution. Several studies in STED microscopy dealt with spherical aberration as it is one of the most common aberration modes. By simply using a glycerol objective and its correction collar to correct for spherical aberrations, Urban *et al*. pushed the imaging depth of STED microscopy up to 120 μm (Urban *et al*. 2011). Angibaud *et al*. used a high refractive index mounting medium (CFM3) as a clearing reagent for fixed samples, which greatly increased the penetration depth and performance of STED microscopy (Angibaud *et al*. 2020).

As an SLM is commonly used to provide phase masks for creating the intensity minima in depletion beams, it is most suitable to correct for the aberrations in the depletion beam path. Lenz *et al*. implemented a non-iterative approach to correct the aberrations in the depletion beam path by generating a look-up table considering the depth and the specific refractive index under the study (Lenz *et al*. 2014). Similarly, Bancelin *et al*. measured the aberrations from agarose beads samples as a function of depth and used them as prior information to correct the aberrations when imaging living brain tissue (Fig. 5B) (Bancelin *et al*. 2021). Although these non-iterative approaches are simpler and faster, they tend to work well only in certain scenarios. One challenge in sensorless AO-STED is the lack of a universal image quality metric that works reliably in different situations. For example, while the image brightness is an effective metric for many kinds of microscopy techniques, it is not quite suited for STED as the stimulated emission is not detected, resulting in a dimmer image. Thus, several groups introduced iterative strategies based on combined image quality metrics (brightness and sharpness) that allow correction for both system-induced and sample-induced aberrations in 3D-STED microscopy (Gould *et al*. 2012; Zdankowski *et al*. 2019; Zdankowski *et al*. 2020). Using the same metric, Gould *et al*. demonstrated the use of an SLM for automatic alignment of the depletion focus to the excitation focus in both 2D- and 3D-STED microscopes (Gould *et al*. 2013). If placed in the common beam path, the SLM can be used to correct for the aberrations in the excitation as well (Gorlitz *et al*. 2018). Using both SLM and DM, Patton *et al*. corrected for the aberrations in all three beam paths together, which permits effective 3D-STED imaging of complex structures in drosophila brains (Patton *et al*. 2016). It should be noted that this metric can be used for aberration correction in other microscopy techniques. Hao *et al*. applied the same strategy to a STED microscope with a 4Pi configuration, achieving sub-50-nm isotropic 3D resolutions in tissues (Hao *et al*. 2021). Recently, Antonello *et al*. proposed a novel wavelet-based metric to interrogate the aberration effect at different scales and demonstrated its performance on a STED microscope (Antonello *et al*. 2020a).

STED is naturally compatible with multiphoton excitation, which is the preferred modality for imaging deep in scattering tissues. Velasco *et al*. built a 2PE-STED microscope by combining 3D-STED with 2PE, red-emitting organic dyes, and WFS-based aberration correction (Velasco *et al*. 2021). They demonstrated aberration-corrected 3D super-resolution imaging at 62-μm depth in fixed mouse brain tissue (Fig. 5C) and 76-μm depth in living mouse brain (Fig. 5D).

## DISCUSSIONS

Aberration correction in microscopy with AO is still a fast-growing field. Established AO methods are being combined with other techniques to improve the aberration correction accuracy and speed, indicating that AO correction is entering the application phase.

Deep learning is a rapidly emerging technique that has been applied to almost every corner of microscopy (Belthangady *et al*. 2019; Tian *et al*. 2021). Recently, it has been introduced to aberration correction in fluorescence microscopy. Convolutional neural networks (CNNs) have been employed to estimate the aberrations from an intensity image (Nishizaki *et al*. 2019; Saha *et al*. 2020) or a SH-WFS pattern (Hu *et al*. 2019; Hu *et al*. 2021). Additionally, CNNs were applied to aberration correction in super-resolution microscopy for accurate localization of single molecules in SMLM (Zhang *et al*. 2018), recovery of the doughnut-shaped focus in STED microscopy (Zhang *et al*. 2019), and phase prediction in SIM (Zheng *et al*. 2021). With the fast evolving in the deep learning field, we expect to see robust and effective AO correction with CNNs in super-resolution microscopy in the foreseeable future.

Most AO techniques apply phase correction in the conjugated pupil plane of the objective lens, which is most straightforward and easy to implement. However, pupil AO may not provide optimal correction over a large field of view with spatially variable aberrations, in which cases another technique that involves placing the AO device conjugate to the main source of aberrations, called conjugate AO, has its advantages (Mertz *et al*. 2015). Applying conjugate AO to multiphoton neuroimaging, Park *et al*. demonstrated dynamic imaging of neural dendrites and microglia dynamics through extremely turbid biological tissue, intact mouse skulls, over an extended corrected field of view (Park *et al*. 2015). Moreover, Park *et al*. developed a multi-pupil AO strategy to expand the correction area by nine-fold using a multifaceted prism array (Park *et al*. 2017).

The potential of AO in microscopy has not yet been fully unleashed. With the maturing of AO techniques that allow imaging deeper in tissues, we expect to see high-order aberration correction or scattering correction to become more important. Although scattering correction and aberration correction share some basic principles, scattering correction typically requires adaptive optical devices with significantly greater degrees of freedom as well as more sophisticated image metrics and algorithms, which is beyond the scope of this review. Besides aberrations, the imaging quality and resolutions of fluorescence microscopy are also affected by other factors such as photobleaching, mechanical stabilities, and detector noise. However, we believe that with the development of brighter dyes and more sensitive detectors, AO-assisted fluorescence microscopy will play an increasingly important role in interrogating cutting-edge biological questions.

## Conflict of interest

Jingyu Wang and Yongdeng Zhang declare that they have no conflict of interest.
